# Supplementary figures and images for: Fatty acid oxidation participates in resistance to nutrient-depleted environments in the insect stages of Trypanosoma cruzi
Source: PLoS Pathog. 2021 Apr 5;17(4):e1009495. doi: 10.1371/journal.ppat.1009495 (PMC8049481; doi:10.1371/journal.ppat.1009495)

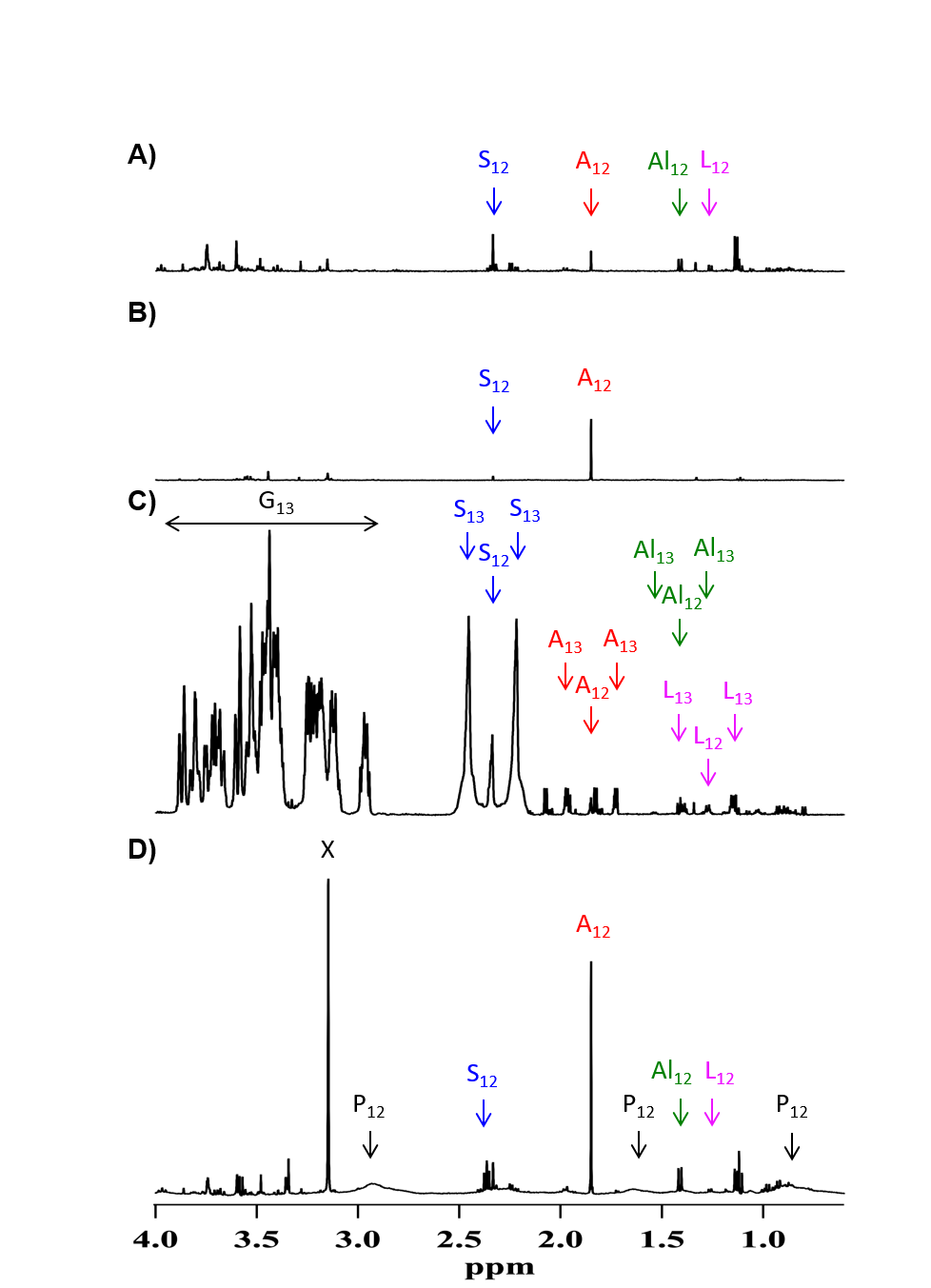

Supplement: S1 Fig — The metabolic end products (succinate, acetate, alanine and lactate) excreted by the epimastigote cells that were incubated after 6 h in PBS (A), PBS after 16 h of starvation without (B) or with D-[U-13C]-glucose (C) or palmitate (D) were determined by 1H-NMR. Each spectrum corresponds to one representative experiment from a set of at least 3. A part of each spectrum ranging from 0.5 ppm to 4 ppm is shown. The resonances were assigned as indicated: A12, acetate; A13, 13C-enriched acetate; Al12, alanine; Al13, 13C-enriched alanine; G13, 13C-enriched glucose; L12, lactate; L13, 13C-enriched lactate; P12, palmitate; S12, succinate; and S13, 13C-enriched succinate. (TIF) [file ppat.1009495.s001.tif]

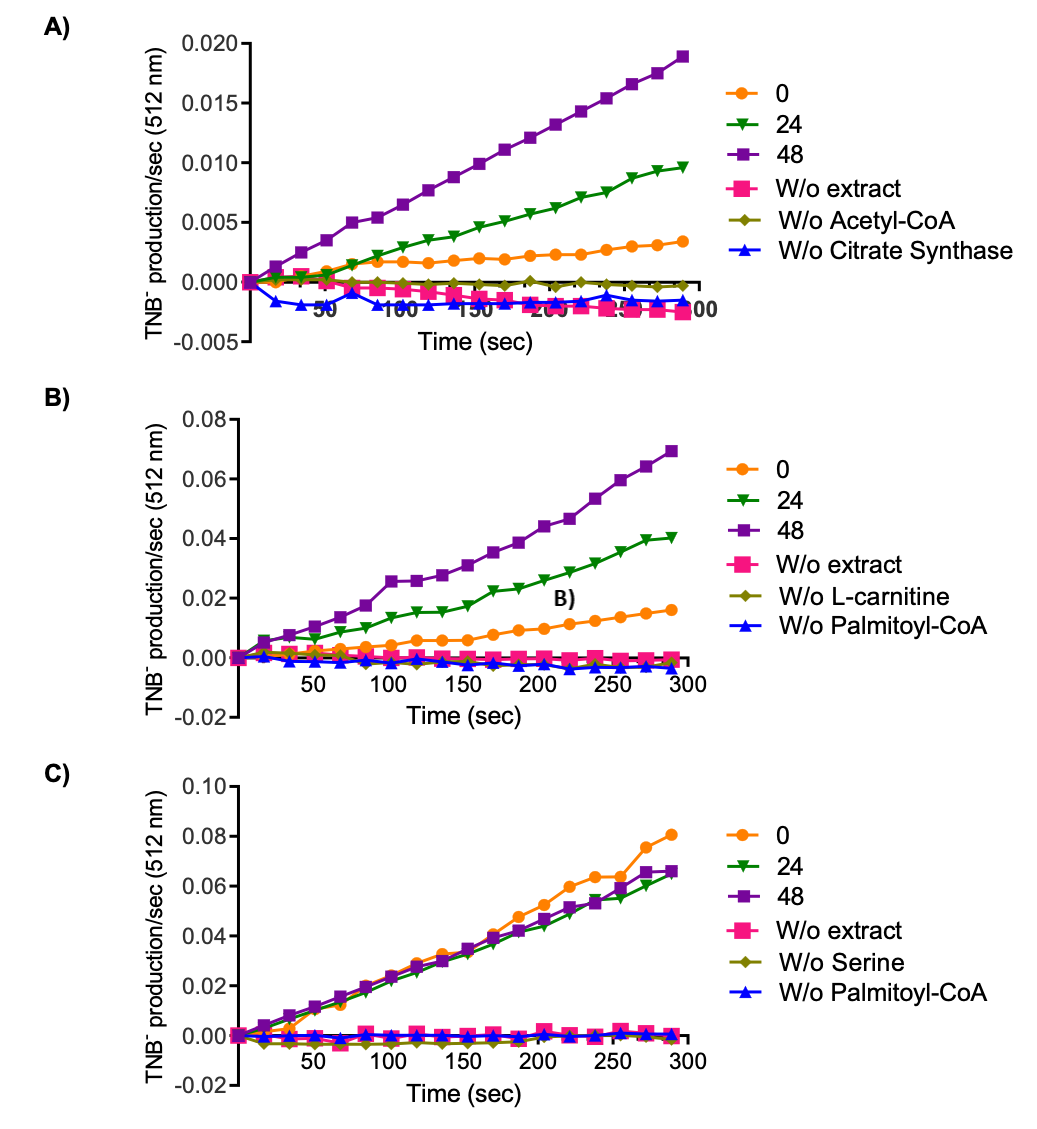

Supplement: S2 Fig — A) (ACC) acetyl-CoA carboxylase, B) (CPT1) carnitine-palmitoyltransferase, and C) (SPT) serine palmitoyltransferase. All the activities were measured in cell-free extracts of epimastigote forms at different moments of the growth curve as indicated in the main text. All the measurements were performed in triplicates. (TIF) [file ppat.1009495.s002.tif]

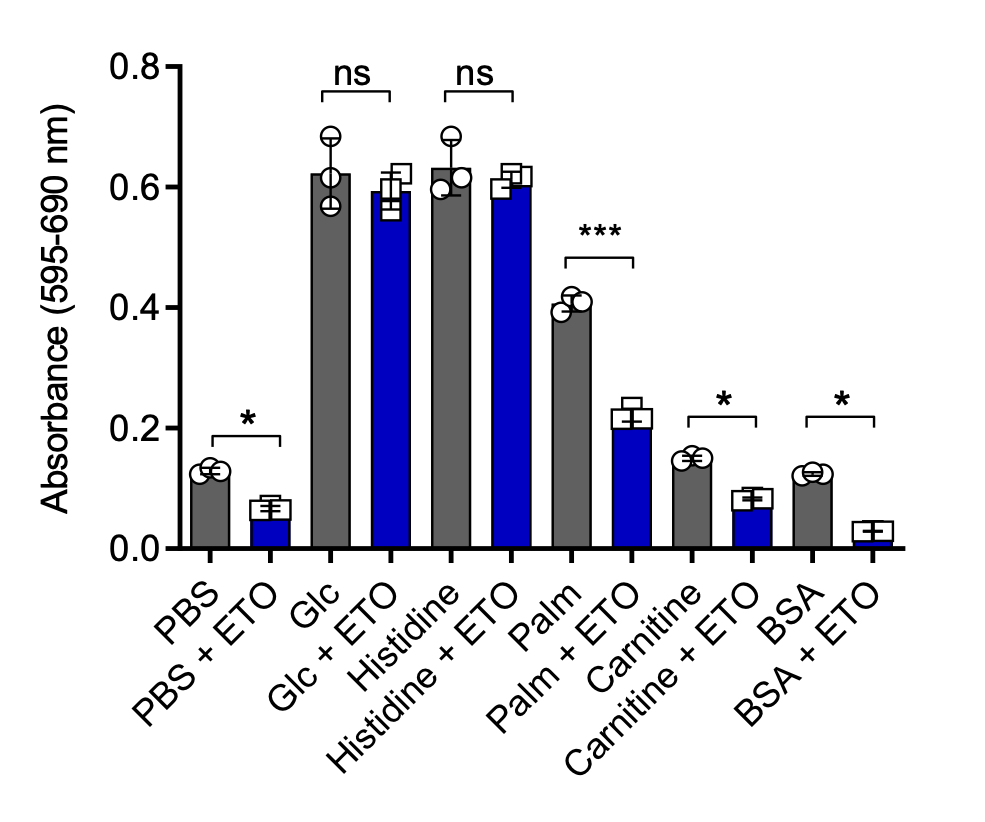

Supplement: S3 Fig — Well-known FAO inhibitors such as valproic acid [57], trimetazidine [58,59] and β-hydroxybutyrate [60], were assayed for their effect on the proliferation of T. cruzi epimastigotes. None of the compounds at the tested concentrations inhibit the epimastigotes proliferation. We used the compounds at the higher concentration tested for epimastigotes proliferation to assay their ability to inhibit FAO by 14CO2 trapping using U-14C-palmitate as a substrate. As observed, none of these compound inhibited the 14CO2 production from palmitate, confirming that they are not inhibiting FAO in T. cruzi. The compounds were assayed at concentrations between 0.1 and 1000 μM. For positive controls of dead cells, a combination of antimycin (0.5 μM) and rotenone (60 μM) were used. The maximum concentration tested for these compounds does not diminish CO2 liberation from FAO. A) Valproic Acid (AV). B) Trimetazidine (TMZ). C) β-hydroxybutyrate (βHOB). (TIF) [file ppat.1009495.s003.tif]

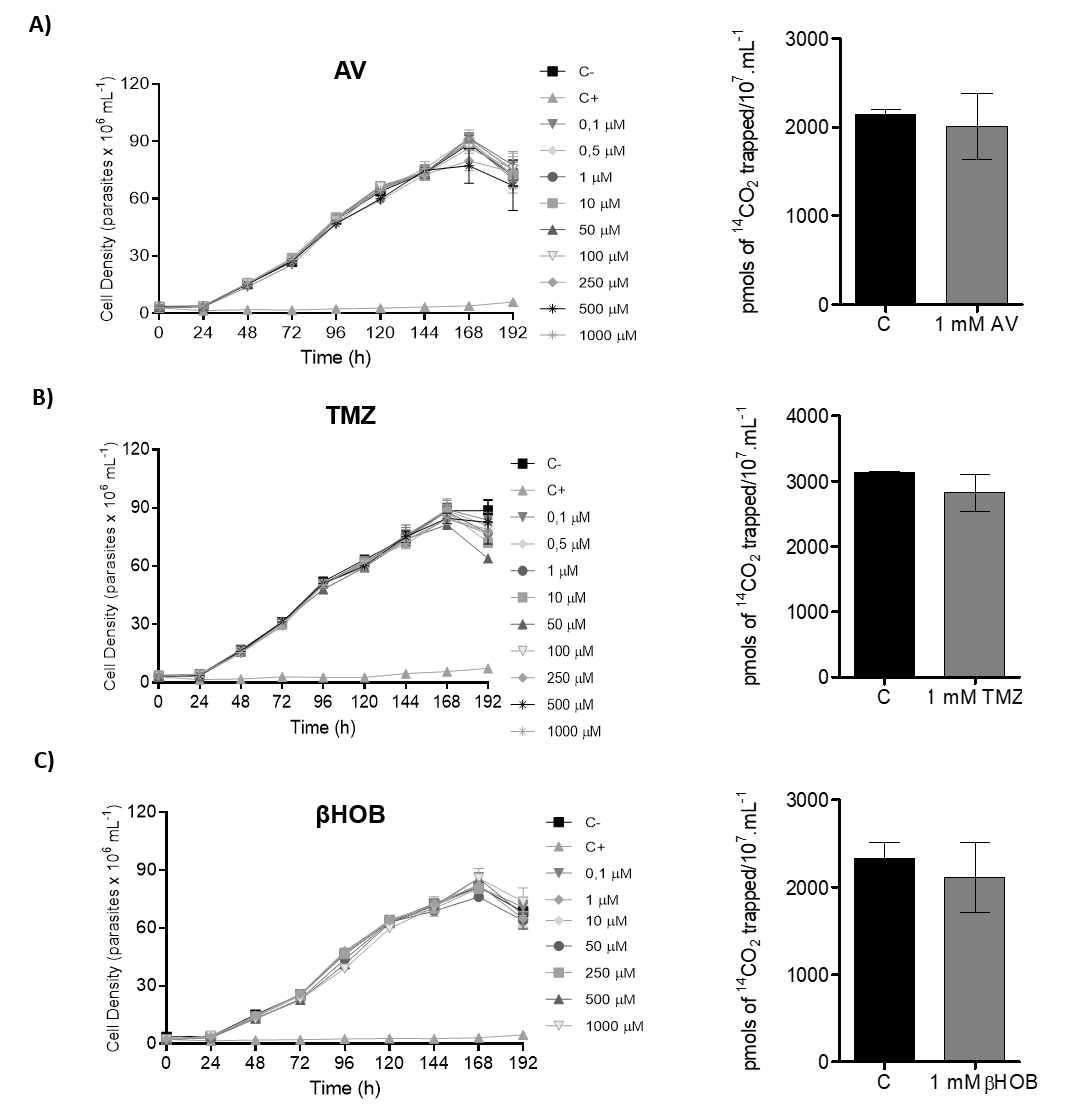

Supplement: S4 Fig — To confirm that ETO is acting on FAO and to rule out that our findings are due to off-target effects that can happenas at concentrations of up to 200 μM [61,62] we assayed its effect in the presence of non-lipidic carbon sources. The parasites were incubated for 24 h in PBS (negative control), 0.1 mM palmitate supplemented with BSA, 5.0 mM histidine, 5 mM glucose, 0.1 mM carnitine and BSA without adding palmitate in the presence (or not) of 500 μM ETO. As expected, ETO treatment did not affect the viability of cells incubated in glucose or histidine but did affect the viability of the cells incubated with palmitate or carnitine. Surprisingly, we also observed an ETO effect on parasites under metabolic stress, such as those incubated with PBS or BSA. This finding could be explained by the fact that under metabolic stress, the parasite mobilizes and consumes its internal lipids. The viability of these cells was inferred from by measuring the total reductive activity using MTT assays after 24 h. (TIF) [file ppat.1009495.s004.tif]

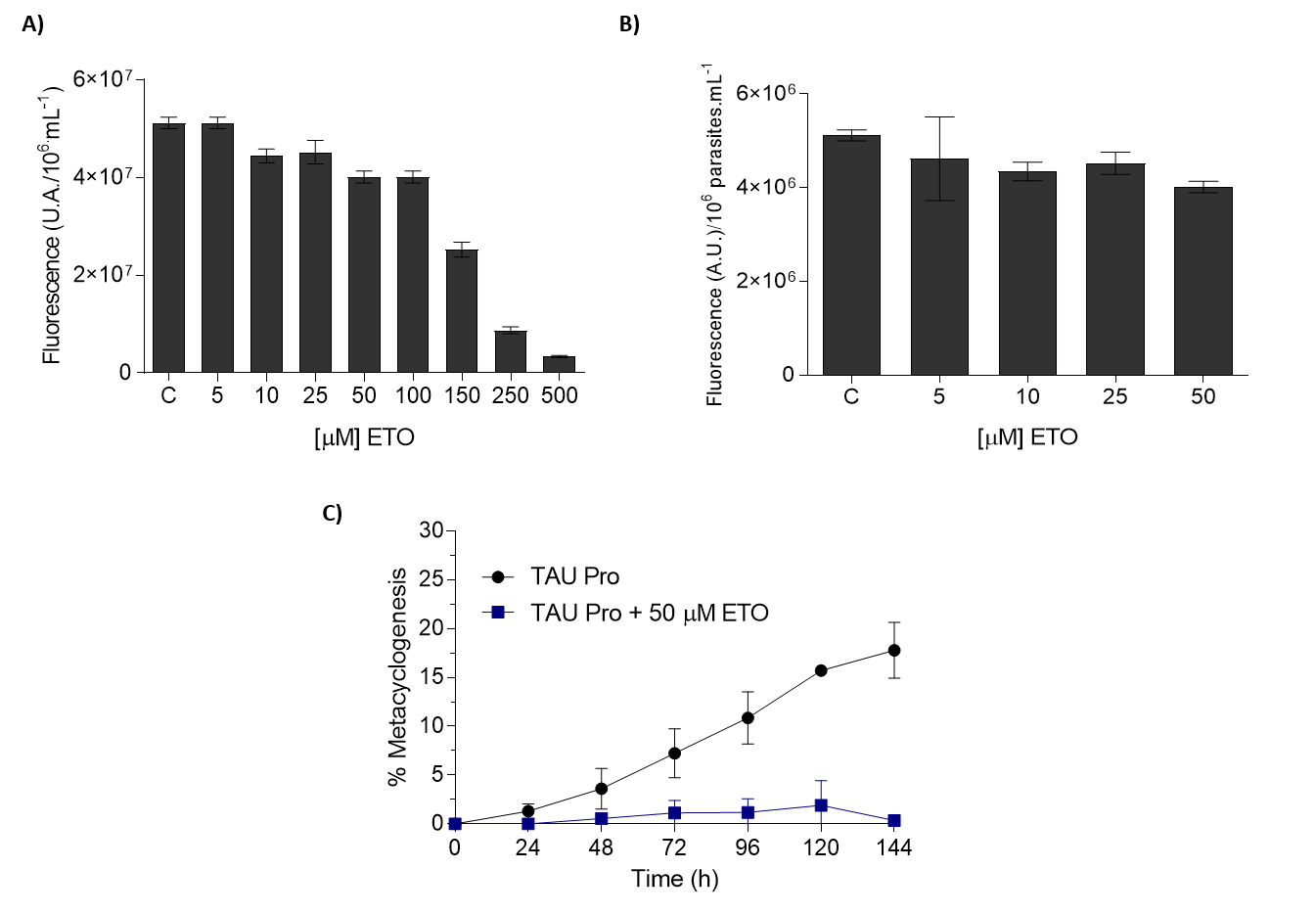

Supplement: S5 Fig — We assassed the viability of parasites during metacyclogenesis in the presence or not of ETO. Stationary epimastigotes in TAU-3AAG media were treated with different concentrations of ETO (5 to 500 μM for 24 hs, panel A, or 5 to 50 μM for 120 h, panel B). The viability of these cells was inferred by measuring the total reductive activity using an Alamar blue assay [63]. In addition, the parasites were maintained during metacyclogenesis in the presence of 50 μM ETO (or not, control) and differentiation was followed up by daily counts, based on the percentage of metacyclic trypomastigotes collected in culture supernatant (panel C). Considering that TAU-3AAG contains glucose in its composition, we performed an in vitro metacyclogenesis using only proline as a metabolic inducer [64]. As observed, even in the absence of glucose, ETO treatment affects metacyclogenesis. (TIF) [file ppat.1009495.s005.tif]
